# A Host Transcriptional Signature for Presymptomatic Detection of Infection in Humans Exposed to Influenza H1N1 or H3N2

**DOI:** 10.1371/journal.pone.0052198

**Published:** 2013-01-09

**Authors:** Christopher W. Woods, Micah T. McClain, Minhua Chen, Aimee K. Zaas, Bradly P. Nicholson, Jay Varkey, Timothy Veldman, Stephen F. Kingsmore, Yongsheng Huang, Robert Lambkin-Williams, Anthony G. Gilbert, Alfred O. Hero, Elizabeth Ramsburg, Seth Glickman, Joseph E. Lucas, Lawrence Carin, Geoffrey S. Ginsburg

**Affiliations:** 1 Institute for Genome Sciences and Policy, Duke University Medical Center, Durham, North Carolina, United States of America; 2 Division of Infectious Diseases, Duke University Medical Center, Durham, North Carolina, United States of America; 3 Durham Veteran’s Affairs Medical Center, Durham, North Carolina, United States of America; 4 Department of Electrical and Computer Engineering, Duke University, Durham, North Carolina, United States of America; 5 National Center for Genome Resources, Santa Fe, New Mexico, United States of America; 6 Center for Computational Biology and Bioinformatics, University of Michigan, Ann Arobor, Michigan, United States of America; 7 Retroscreen Virology, London, United Kingdom; 8 Duke Human Vaccine Institute, Duke University Medical Center, Durham, North Carolina, United States of America; 9 Department of Emergency Medicine, University of North Carolina-Chapel-Hill, Chapel Hill, North Carolina, United States of America; The University of Hong Kong, Hong Kong

## Abstract

There is great potential for host-based gene expression analysis to impact the early diagnosis of infectious diseases. In particular, the influenza pandemic of 2009 highlighted the challenges and limitations of traditional pathogen-based testing for suspected upper respiratory viral infection. We inoculated human volunteers with either influenza A (A/Brisbane/59/2007 (H1N1) or A/Wisconsin/67/2005 (H3N2)), and assayed the peripheral blood transcriptome every 8 hours for 7 days. Of 41 inoculated volunteers, 18 (44%) developed symptomatic infection. Using unbiased sparse latent factor regression analysis, we generated a gene signature (or factor) for symptomatic influenza capable of detecting 94% of infected cases. This gene signature is detectable as early as 29 hours post-exposure and achieves maximal accuracy on average 43 hours (p = 0.003, H1N1) and 38 hours (p-value = 0.005, H3N2) before peak clinical symptoms. In order to test the relevance of these findings in naturally acquired disease, a composite influenza A signature built from these challenge studies was applied to Emergency Department patients where it discriminates between swine-origin influenza A/H1N1 (2009) infected and non-infected individuals with 92% accuracy. The host genomic response to Influenza infection is robust and may provide the means for detection before typical clinical symptoms are apparent.

## Introduction

Infectious disease diagnostics traditionally rely heavily on pathogen detection [1], [2], [3]. However, the development of reproducible means for extracting RNA from whole blood, coupled with advanced statistical methods for analysis of complex datasets, has created the possibility of classifying infections based on host gene expression profiling. We recently developed a robust whole blood mRNA expression classifier for human respiratory viral infection at the time of *maximal* symptoms using data from three human viral challenge cohorts (rhinovirus, respiratory syncytial virus, and H3N2 influenza A) [4]. Sparse latent factor analysis of peripheral blood mRNA expression data revealed a pattern of gene expression common across symptomatic individuals from all viral challenges [4]. Furthermore, an analysis of publically available peripheral blood-based gene expression data indicated that the respiratory viral signature could distinguish patients with symptomatic viral infections from those with bacterial infections as well as from healthy controls [4], [5].

The emergence of pandemic H1N1 influenza in 2009 highlights the need for new approaches to diagnosis of respiratory tract infections. A diagnostic test that could identify patients before the onset of symptoms (but after exposure) who will later become ill would be an indispensible tool for guiding individual treatment decisions when antiviral supplies may be limited. Furthermore, these early results may forecast epidemic/pandemic spread, potentially mitigating pandemic intensity [6]. Although previous studies with dengue, melioidosis, tuberculosis, candidiasis, and sepsis have focused on diagnosis in patients as they present with active disease [7], [8], [9], [10], [11], [12], we utilized human influenza challenge cohorts with a defined inoculation event coupled with dense serial sampling to explore the ability of modern genomic and statistical techniques to accurately classify individuals with multiple subtypes of influenza infection as early as possible following viral exposure. Through this method, we have demonstrated the potential for a robust host gene response signature in pre-symptomatic human infection and suggest the utility of this approach for detecting pandemic H1N1 infection in an acute care setting.

## Results

### Healthy Volunteers Demonstrate Variable Clinical Responses to Inoculation with Seasonal Influenza H1N1 and H3N2

**Figure 1:**
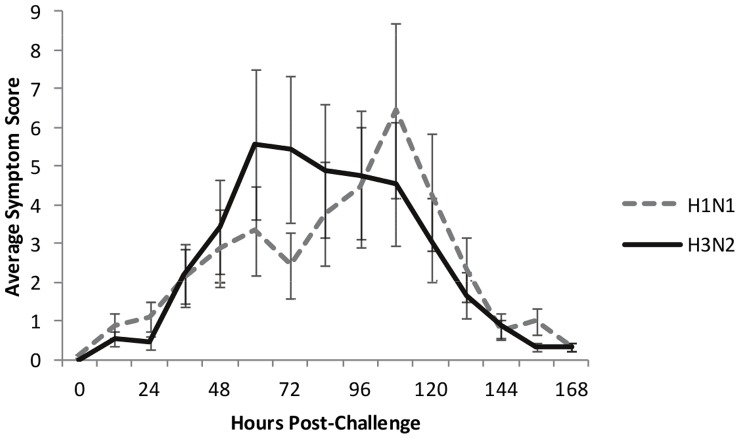
Clinical response to viral challenge. Average symptom scores over time of individuals with both clinical and microbiologically confirmed infection (symptomatic-infected) following experimental viral inoculation with H1N1 (blue) and H3N2 (red).

For the H1N1 challenge we inoculated 24 volunteers age 20–35 with influenza A (A/Brisbane/59/2007). Nine (38%) of the 24 inoculated subjects developed symptoms consistent with viral upper respiratory infection with confirmed shedding of challenge virus (Fig. 1). This infection rate is similar to previous viral challenge studies [13], and occurs despite similar patient profiles, vaccination history, and baseline influenza hemagglutination and neutralization titers (Sup Tables s1 and s2). Subjects exhibited variability of time to initiation of symptoms as well as maximal severity of symptoms achieved (Fig. s1), but symptom onset began an average of 61.3 hours after inoculation (range 24–108 hrs, median 72 hrs). Subjects who became ill experienced maximal symptoms on average 102.7 hours after inoculation (range 60–120 hours, median 108 hrs). For symptomatic subjects, the average total 5 day symptom score was 19.7 (range 6–34) with an average daily peak of 7.4 (range 4–13, Table s3).

For the H3N2 challenge (A/Wisconsin/67/2005) reported previously [4], [14], we inoculated 17 volunteers (mean age 27 years; range 22–41 years). For the 9 (53%) symptomatic-infected subjects, symptom onset began earlier than in the H1N1 challenge (Fig. 1) at an average of 49.3 hours after inoculation (range 24–84 hours, median 48 hrs). Subjects who became ill experienced maximal symptoms on average 90.6 hours after inoculation (range 60 to 108 hours, median 96 hours). For these subjects the average total 5 day symptom score was 21.1 (range 6–43) with an average daily peak of 7.3 (range 2–13).

For both challenge studies, only those individuals achieving both clear clinical and virologic endpoints were analyzed as true influenza ‘infection’ (see Methods, Table s3). In our challenge studies there were four major outcome groups despite historical and immunologic screening and similar inoculations [13]. Most individuals fall within our two analysis groups – those who are symptomatic-infected or asymptomatic-uninfected. However, a few individuals demonstrate mixed phenotypes and are either symptomatic-*un*infected (symptoms but no viral shedding detected, see Methods) or *a*symptomatic-infected individuals (never symptomatic but clear viral shedding on multiple days (Table s3). We have focused this analysis on those subjects with the clear phenotypes of ‘infected’ and ‘uninfected’ (see Methods for phenotyping criteria). The development of biomarkers for asymptomatic-infected and symptomatic-uninfected and a understanding their underlying biology would be invaluable, and could potentially inform our ability to forecast and track epidemics. However, the numbers of such individuals from the current studies are insufficient for meaningful analysis at this time.

**Figure 2:**
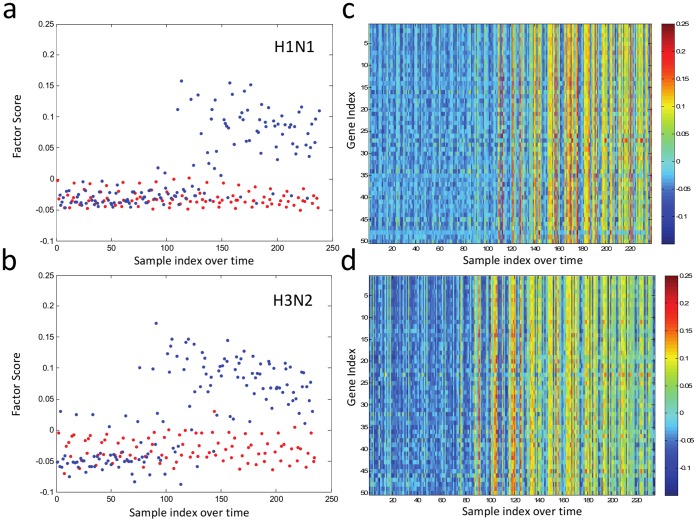
Gene expression signatures expressed through factor scores. An influenza gene expression signature, or factor, evolves over time in symptomatic individuals (blue dots) and distinguishes between symptomatic and asymptomatic individuals (red dots) for both H1N1 (A) and H3N2 (B) viruses at later time points. Heat maps of the top 50 genes in the discriminative factor for H1N1 (c) and H3N2 (d) as they develop over time are shown.

**Influenza-induced host gene expression groups into unbiased time-evolving factors** Whole blood RNA was isolated from each individual every 8 hours from inoculation through day 7 and assayed by Affymetrix U133a 2.0 human microarrays. Co-expressed gene transcript factors were generated through sparse latent factor regression analysis to provide an unbiased (unlabeled) examination of gene expression [15]. This methodology specifically selects gene ‘factors’, with each factor effectively defining a specific, limited subset of genes that are up- or down-regulated in a given condition. Sparse latent factor regression analysis permits an unbiased selection of these co-regulated genes while simultaneously filtering the tremendous number of genes tested into smaller, more manageable, biologically connected subsets (see Methods). Based upon the quantitative level of over- or under-expression of the individual genes in a factor, a factor score is computed for a given factor in a given sample at a given time. In each individual, the factor score for each group of co-expressed genes evolve as they progress through the various stages of disease (Fig. 2a, b). Furthermore, within each factor, the individual genes themselves exhibit variable expression over time (Fig. 2c, d, Fig. s2), and therefore each gene’s individual contribution to a single factor score continuously evolves, highlighting the complexity of the temporal dynamics of the host response to influenza challenge. The factor score provides a coherent representation of the aggregate of these co-expressed genes at a given time-point allowing for a more manageable means of expressing biologically relevant genomic variance over time.

### A Whole Blood RNA-based Gene Signature Differentiates Symptomatic Influenza A H1N1 or H3N2 Infection from Asymptomatic Individuals

Similar to our previous work [4], in each challenge a single factor emerged as best able to discriminate symptomatic-infected subjects from asymptomatic-uninfected subjects at the time of maximal symptoms (Fig. 2). We derived this gene signature or “Influenza Factor” individually for both H1N1 and H3N2 and found that the signature is highly conserved across the two different viruses. For H1N1, the derived factor correctly identifies our phenotypically confirmed individuals exposed to H1N1 as symptomatic-infected or asymptomatic-uninfected with only a single misclassification, whereas the H3N2 factor correctly identifies 100% of individuals exposed to H3N2 with a confirmed phenotype.

The performance of two separate clinical challenge studies with closely related viruses permits the validation of the independently derived gene signatures by testing them on the subjects from the alternate study. When the factor loadings for H1N1 are applied to the subjects from the H3N2 study, the H1N1 factor is capable of accurately discriminating between symptomatic-infected or asymptomatic-uninfected H3N2 subjects 100% of the time (Fig. s3). Similarly, when applied to the H1N1 data set, the H3N2 factor correctly identifies 93% (14/15) of the subjects in the H1N1 study as symptomatic-infected or asymptomatic-uninfected. Thus, each of the independently derived factors for H1N1 or H3N2 performs well when applied to a completely separate data set comprised of individuals with a similar yet distinct pathogen.

### Discriminatory Factors for H1N1 and H3N2 are Similar and Include Genes Involved in the Antiviral Response

The gene signatures derived independently for the two different strains of influenza are highly similar, sharing 44 out of the top 50 genes (88%, Table s4). However, the importance of these few disparities is unclear, as the discordant genes are not sufficient to allow for differentiation between the two viruses in our analysis. When compared to our previous work with HRV and RSV, the Influenza Factor shares only 65–69% of its genes with factors describing infection with these other respiratory viruses, suggesting both common ‘viral URI’ pathways as well as some degree of etiologic specificity. The majority of the top 50 predictive genes contained in each factor are known to characterize host response to viral infection, and include RSAD2, the OAS family, multiple interferon response elements, the myxovirus-resistance gene MX1, cytokine response pathways and others [16], [17], [18]. Many (but not all) of the components of these gene sets can be combined into networks that putatively describe interactions between factor-derived genes in canonical inflammatory and antiviral pathways (Fig. s4). Furthermore, the high degree of similarity and cross-applicability of the two signatures permit the mathematical imputation of a combined “Influenza Factor” that retains the discriminatory characteristics of the individual factors when applied to both cohorts (Fig. s5).

### The Influenza Factor Tracks Closely with Symptom Scores over Time and is Capable of Identifying Symptomatic-infected Individuals Before the Time of Maximal Illness

**Figure 3:**
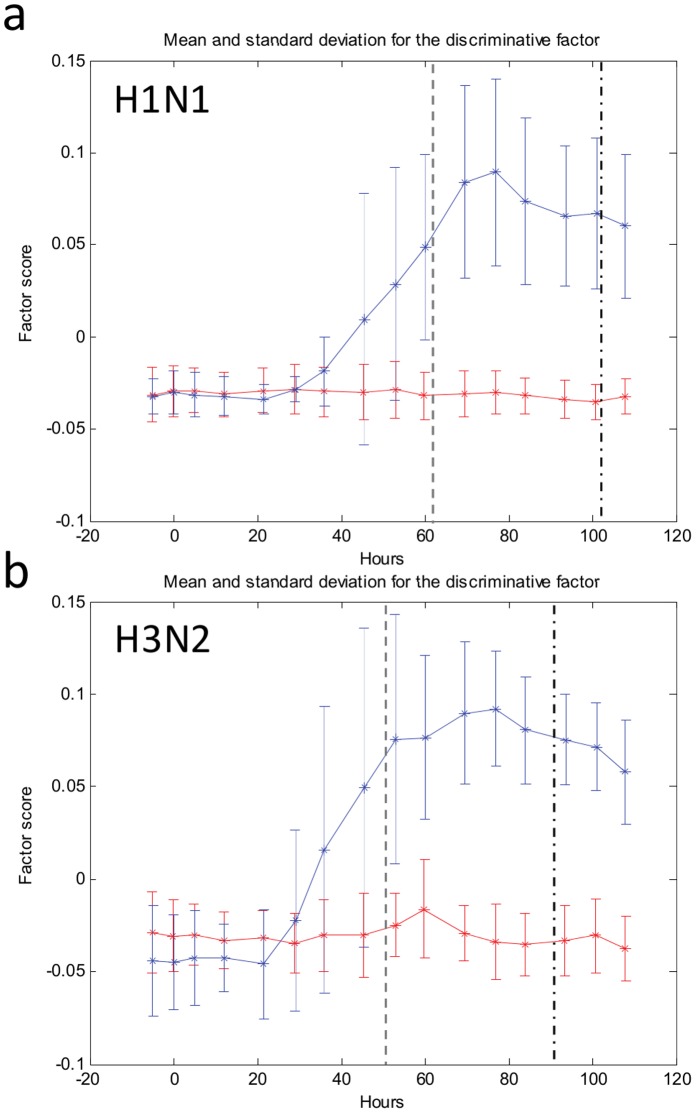
Gene expression signature trajectory over time. The magnitude of the Influenza Factor varies from inoculation through resolution of disease, for both H1N1 (A) and H3N2 (B) patients. The average factor score at each timepoint for both symptomatic (blue) and asymptomatic (red) individuals are shown. The average time of symptom onset (gray dashed line) and maximal symptoms (black dashed line) are depicted.

We next sought to define the clinical performance of the Influenza Factor over time. Just as symptom scores, time of peak symptoms, and symptom progression vary over time between individuals (Fig. 1), the rise and fall of the gene expression based factor score varies as well, and a common factor trajectory can be mathematically imputed for all symptomatic subjects (Fig. 3a–b). The trajectory of the Influenza Factor for symptomatic, infected individuals first begins to diverge from asymptomatic, uninfected individuals at 35–40% of the elapsed time between inoculation and the time of maximal symptoms for each individual (38 hours post-inoculation for H1N1 and 29 hours for H3N2, Fig. 3a–b). Even in this controlled challenge study among young, healthy individuals, we find variability in this temporal relationship, similar to the individual variability seen with symptom scores. In most symptomatic individuals, the rise, peak, and fall of the factor score trajectory tends to mimic in character but precede the changes in the clinical score (Fig. s6). Even with this variability and relatively limited sample size (9 symptomatic-infected individuals in each study), the symptomatic-infected factor trajectory diverges by 53 hours (H3N2, p-value = 0.005) and 60 hours (H1N1, p-value = 0.003) post-inoculation.

We developed Receiver Operating Characteristic (ROC) curves at each time point to visualize the ability of the Influenza Factor to discriminate between symptomatic- infected and asymptomatic-uninfected subjects (Figure s7). For H3N2 infection, the factors can distinguish between symptomatic and asymptomatic individuals with a sensitivity of 89% without false positives at 53 hours post-exposure. By 69 hours post-inoculation the sensitivity is increased to 100%. For H1N1, this occurs slightly later but by 60 hours post-exposure the Influenza Factor demonstrates a sensitivity of 89% without false positives. These time points that the gene signature first effectively discriminates symptomatic vs. asymptomatic subjects usually precede or coincide with the time of average first symptom onset (49 hrs for H3N2 and 61 hours for H1N1), and occur well before clinically significant symptoms (38 hours before maximal symptoms for H3N2 and 43 hours for H1N1).

### The Influenza Factor Accurately Identifies Pandemic 2009 H1N1 Infections in a Clinical Cohort

**Figure 4:**
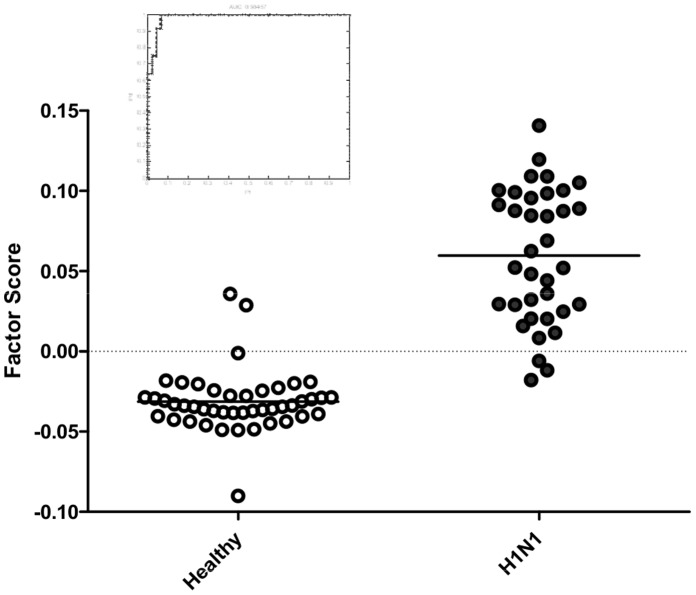
Validation of the Influenza Factor in a real-world cohort of individuals presenting with confirmed swine-origin 2009 A/H1N1 infection. The Influenza Factor scores distinguish individuals with RT-PCR proven H1N1 infection (•) from healthy individuals (○) as demonstrated both by factor score and by ROC curve for healthy vs. H1N1 (insert, AUC 0.98).

In order to assess the validity of the experimentally derived Influenza Factor to perform in a free-living (non-experimental) setting we used a cohort of individuals enrolled during the 2009–10 Influenza season. At that time, we identified 36 individuals who presented to the Duke University Hospital emergency department with symptomatic H1N1 infection (confirmed by RT-PCR), and 45 healthy controls. Peripheral blood RNA samples were obtained from the symptomatic individuals at the time of presentation with symptomatic respiratory viral infection. The Influenza Factor was applied to the microarray data derived from the blood RNA samples and correctly identifies 92% (33/36) of the subjects as infected with Novel H1N1, and correctly identified 93% (42/45) of the healthy controls (Fig. 4). Overall, the Influenza Factor performed with an accuracy of 92.3% in the setting of a real-world, independent cohort with pandemic 2009 H1N1 infection.

## Discussion

We performed two independent human viral challenge studies (using influenza H1N1 and H3N2) to define the host-based peripheral blood gene expression patterns characteristic of the response to influenza infection. The results provide clear evidence that a biologically relevant peripheral blood gene expression signature can distinguish influenza infection with a remarkable degree of accuracy across the two strains. We have also defined the performance of the blood gene expression signature over time throughout the complete course of human influenza infection. Furthermore, despite arising from a controlled experimental challenge setting, we demonstrate that an influenza signature is able to accurately identify individuals presenting with naturally-occurring, RT-PCR confirmed H1N1 infection during the 2009 pandemic.

Defining the etiology of clinical syndromes in which infection is suspected remains challenging. Currently available influenza diagnostic tests exhibit highly variable sensitivity, ranging from 53 to 100% in various studies [19], [20]. Importantly, even those with powerful test characteristics such as RT-PCR are dependent upon sampling technique and inclusion of virus-specific components leading to reduced effectiveness with emerging viral strains [21]. In addition to being less susceptible to sampling error, genomic signatures are not viral antigen or nucleic acid-dependent, and unlikely to be as strain-specific as pathogen-based platforms. Therefore, in addition to high sensitivity in the cohorts studied [92% (95% CI 79–99% for 2009 H1N1)], influenza gene signatures have the added potential of being able to identify, in the acute phase of illness, likely cases of infection with emerging influenza strains for which a specific diagnostic platform has yet to be developed and distributed. The nature of challenge studies limits our ability to make direct comparisons to other infected states – however, our previous work has demonstrated that genomic signatures similarly derived from viral challenges are capable of distinguishing upper respiratory viral infection from pneumonia due to *Streptococcus pneumoniae* [*4*] *.* These findings are promising but additional testing of these signatures in other models, including acute human cases of bacterial infection, will need to be performed to better delineate their specificity.

The unique design and frequent sampling involved in two experimental challenge studies has also given us the singular ability to examine the dynamics of temporal development of the genomic responses following exposure to infectious virus. We have shown that when viewed through the lens of the genomic response, it is possible to correctly distinguish individuals as infected or uninfected with influenza well before they have clinically relevant symptoms or would be ill enough to present for clinical evaluation. The potential power of this approach is manifested by full discriminative ability of the genomic signature as early as 53 hours post-viral exposure, at a time when the average clinical score of symptomatic individuals is only 2.4. Symptoms of this nature and severity are clinically vague and would be typical of very mild allergies [22] or even symptoms due to sequelae of chronic smoking [23]. Therefore, genomic analyses demonstrate the potential to identify viral infection either before symptoms emerge or among what otherwise are common, nonspecific upper respiratory symptoms, when early intervention with antiviral medications could have profound impact on both individual symptoms and disease transmission [24], [25], [26]. Furthermore, we show that the overall trajectory of the Influenza Factor tracks closely with symptom scores over time, but also that the observed genomic response tends to significantly precede changes in clinical scores in symptomatic individuals. None of our affected individuals developed severe infection, but the characteristics of the timing and development of these signatures suggest that, similar to recent work with Dengue infections [9], genomic signatures may potentially prove invaluable for predicting clinical outcomes. However, further longitudinal studies with patients who eventually exhibit more severe disease will be required to fully assess this potential.

The nature of the individual components of the genomic response to influenza infection and the biological pathways they represent lend plausibility to this discovery. In particular, interferon stimulated pathways such as those including RSAD2, IRF7, MX1, OAS3, MDA-5, RIG-I and others are incorporated and thought to drive both innate and, to a lesser degree, adaptive immune responses to viral infection [18], [27], [28], [29]. Many of these pathways are consistent with those identified in acutely ill pediatric influenza subjects [30] and recent studies of the genomic response following vaccination with live, attenuated influenza vaccine reported a profile of ‘immune activation’ which shares a number of genes with the Influenza Factor described here [31], [32]. Interestingly, a few genes which consistently feature prominently in the Influenza Factor are not clearly tied to inflammatory or immunologic pathways, and their significance remains unclear. Previously published work with bacterial respiratory infections has yielded quite different genomic results [4], [5] suggesting that some aspects of the host response are specific at least for major classes of pathogens (e.g., viral vs. bacterial). The genomic pathways identified suggest we are largely measuring indicators of the development/amplification of the immune response to the virus similar to previous work [13], [16], [32], and that these indicators parallel (and usually precede) clinical symptom development in time. The immunologic pathways observed in these studies that are known to be commonly activated early on at the primary site of infection (i.e., respiratory epithelium) [29], [33], exhibit relatively delayed appearance in the periphery. This delay seems logical, as early innate responses at the site of infection would be expected to have an initially minor impact on global peripheral gene expression. At very early time-points (<53 hrs following exposure) insufficient numbers of peripheral cells are undergoing the conserved stimulation required to produce a significant change in global gene expression, at least as detected by microarray analysis. This raises the possibility that more sensitive methods of detecting genomic changes, such as individual cell-type sampling or RT-PCR of select genes, will prove to be even more precise at early time points in the evolution of viral infection. Additional work will be essential (and is underway) to further define the nature and biological implications of these data, as well as to work towards development of a more practical means of assaying these changes in the clinical setting, such as RT-PCR of select ‘core’ genes from signatures like the one described herein.

Clearly, great care must be taken when analyzing and applying host genomic data from human challenge studies where the means of transmission of the virus is experimentally designed rather than ‘natural’, and the degree of illness which follows is not always typical of the severity seen in naturally acquired infection in subjects who present for clinical care, even though it does tend to mimic the overall character of natural clinical disease [13]. Hosts in these studies are universally young, healthy individuals at minimal risk for developing severe complications, which may limit the broad applicability of such findings, although this is somewhat mitigated by the strong performance of the gene signatures despite significant clinical variability in infected subjects. It is also important to note that while this type of factor analysis allows for description of conserved biological pathways indicative of influenza infection, a given factor only represents a limited interrelated subset of all genes that are globally up- or down-regulated in response to a given condition, and thus does not describe the entirety of the genomic response.

Despite these limitations, we have for the first time defined the temporal dynamics of a genomic signature driving the host response to influenza infection in humans. These molecular and statistical techniques combined with the ability to longitudinally study exposed human hosts have given us the opportunity to examine periods of human disease which have previously been largely unexplored. Moreover, despite being developed in an experimental challenge model, this host genomic signature performs at a high level of accuracy in the setting of naturally acquired pandemic 2009 H1N1 infection. This work demonstrates that analyses of the temporal development of gene expression signatures shows promise both for creating diagnostics for early detection, as well as providing insight into the biology of the host response to influenza and other pathogens.

## Materials and Methods

### Institutional Review Board Approvals

The Influenza challenge protocols were approved by the East London and City Research Ethics. Committee 1 (London, UK), an independent institutional review board (WIRB: Western. Institutional Review Board; Olympia, WA), the IRB of Duke University Medical Center. (Durham, NC), and the SSC-SD IRB (US Department of Defense; Washington, DC) and were conducted in accordance with the Declaration of Helsinki. All subjects enrolled in viral challenge studies provided written informed consent per standard IRB protocol. Funding for this study was provided by the US Defense Advanced Research Projects Agency (DARPA) through contract N66001-07-C-2024 (P.I., Ginsburg).

### Human Viral Challenges

In collaboration with Retroscreen Virology, Ltd (London, UK), we intranasally inoculated 24 healthy volunteers with influenza A H1N1 (A/Brisbane/59/2007). All volunteers provided informed consent and underwent extensive pre-enrollment health screening, including baseline antibody titers to the specific strains of influenza utilized. After 24 hrs in quarantine, we instilled one of four dilutions (1∶10, 1∶100, 1∶1000, 1∶10000) of 10^7^ TCID_50_ influenza A into bilateral nares of subjects (groups of 4–6 for each dilution) using standard methods. [4] The virus was manufactured and processed under current good manufacturing practices (cGMP) by Baxter BioScience, (Vienna, Austria). At pre-determined intervals (q8h for the first 5d following inoculation), we collected blood into RNA PAXGene™ collection tubes (PreAnalytix; Franklin Lakes, NJ) according to manufacturers’ specifications. We obtained nasal lavage samples from each subject daily for qualitative viral culture and and/or quantitative influenza RT-PCR to assess the success and timing of infection [34]. Blood and nasal lavage collection continued throughout the duration of the quarantine. All subjects received oral oseltamivir (Roche Pharmaceuticals) 75 mg by mouth twice daily as treatment or prophylaxis at day 6 following inoculation. All subjects were negative by rapid antigen detection (BinaxNow Rapid Influenza Antigen; Inverness Medical Innovations, Inc) at time of discharge. Detailed methods of the H3N2 Challenge study have been reported previously [4], [14].

### Clinical Case Definitions

Symptoms were recorded twice daily using a modified standardized symptom score [35]. The modified Jackson Score requires subjects to rank symptoms of upper respiratory infection (stuffy nose, scratchy throat, headache, cough, etc) on a scale of 0–3 of “no symptoms”, “just noticeable”, “bothersome but can still do activities” and “bothersome and cannot do daily activities”. For all cohorts, modified Jackson scores were tabulated to determine if subjects became symptomatic from the respiratory viral challenge. Symptom onset was defined as the first of 2 contiguous days with score of 2 or more. A modified Jackson score of ≥6 over a consecutive five day period was the primary indicator of symptomatic viral infection [36] and subjects with this score and a positive qualitative viral culture or quantitative RT-PCR for at least 2 consecutive days (beginning 24 hours after inoculation) were denoted as ”symptomatic infection” and included in the signature performance analyses. [35], [36], [37]. Subjects were classified as “asymptomatic, not infected” if the symptom score was less than 6 over the five days of observation and viral shedding was not documented after the first 24 hours subsequent to inoculation as above. Standardized symptom scores were tabulated at the end of each study to determine attack rate and time of maximal symptoms. Some subjects in each study (2 H3N2 and 8 H1N1 subjects) demonstrated an overall picture that fell in between these two categories. These individuals were either ‘asymptomatic viral shedders’ (2 H3N2 and 5 H1N1) or ‘symptomatic non-viral shedders’ (0 H3N2 and 3 H1N1). One additional individual in the H1N1 study was excluded due to additional infection acquired during the study. Given the heterogeneity of their overall ‘infected’ status these individuals were not included in performance analyses.

### Pandemic 2009 H1N1 Real-World Cohort

Subjects were recruited from the Duke University Medical Center Emergency Department (DUMC-Level 1 Trauma Center with annual census of 65,000). This study was approved by the Institutional Review Board at each institution and written, informed consent was obtained by all study participants or their legal designates. Subjects were screened between September 1 and December 31, 2009. Subjects were considered for the enrollment if they had a known or suspected influenza infection on the basis of clinical data at the time of screening and if they exhibited two or more signs of systemic inflammation (SIRS) within a 24-hour period. Subjects were excluded if <18 years old, if they had an imminently terminal co-morbid condition, if they had recently been treated with an antibiotic for a viral, bacterial, or fungal infection, or if they were participating in an ongoing clinical trial.

Trained study coordinators at each site reviewed and abstracted vital signs, microbiology, laboratory, and imaging results from the initial ED encounter and at 24-hour intervals if patient was admitted. Following hospital discharge, research personnel abstracted the duration of hospitalization, length of ICU stay, in-hospital mortality, timing and appropriateness of antimicrobial administration, and microbiologic-culture results from the medical record. In addition to residual respiratory samples collected as part of routine care, an NP swab was collected from each enrolled subject. Total nucleic acids were extracted from nasal swab or wash isolates with the EZ1 Biorobot and the EZ1 Virus Mini Kit v2.0 (Qiagen). 2009 H1N1 virus was confirmed in 20 ul detection reactions, Qiagen One-Step RT-PCR (Qiagen) reagents on a LightCycler v2.0 (Roche) using the settings and conditions recommended in the CDC Realtime RTPCR (rRTPCR) Protocol for Detection and Characterization of Swine Influenza (version 2009). The primers and probes were as described in the CDC protocol and obtained from Integrated DNA Technologies. We included mRNA expression data obtained concurrently with the H1N1 cohort from 45 gender-matched, healthy controls.

### RNA Purification and Microarray Analysis

For each challenge, we collected peripheral blood at 24 hours prior to inoculation with virus (baseline), immediately prior to inoculation (pre-challenge) and at set intervals following challenge. RNA was extracted at Expression Analysis (Durham, NC) from whole blood using the PAXgene™ 96 Blood RNA Kit (PreAnalytiX, Valencia, CA) employing the manufacturer's recommended protocol. Complete methodology can be viewed in the Methods S1. Hybridization and microarray data collection was also performed at Expression Analysis (Durham, NC) using the GeneChip® Human Genome U133A 2.0 Array (Affymetrix, Santa Clara, CA). Microarray data used for this study will be deposited in GEO prior to publication.

### Statistical Analyses

Following RMA normalization of raw probe data, sparse latent factor regression analysis was applied to each dataset [38], [39], [40], [41]. This reduces the dimensionality of the complex gene expression array dataset assuming that many of the probe sets on the expression array chip are highly interrelated (targeting the same genes or genes in the same pathways). Dimension reduction is performed by constructing factors (groups of genes with related expression values). These factors are used in a sparse linear regression framework to explain the variation seen in all of the probe sets. By default, most of the coefficients in this linear regression are zero. Thus, a small number (e.g., 50) of factors explain variation seen in any single dataset.

Factor loadings are defined as the coefficients of the factor regression, and, to explore the biological relevance any particular factor, we examine the genes that are "in" that factor – the genes that show significantly non-zero factor loadings. “Factor scores" are defined as the vector that best describes the co-expression of the genes in a particular factor. Both factor loadings and factor scores are fit to the data concurrently, and the full details of the process can be found in the supplementary statistical analysis section. While 50 factors were used for the results reported here, we also considered 20, 30 and 40, with minimal effect on the significant factor loadings. Notably, the initial models built to determine factors that distinguish symptomatic infected individuals from asymptomatic individuals were derived using an unsupervised process (i.e., the model classified subjects based on gene expression pattern alone, without *a priori* knowledge of infection status).

Our statistical model is unsupervised, and thus seeks to describe the statistical properties of the expression data without using labeled data. Such unsupervised algorithms may uncover statistical characteristics that distinguish symptomatic and asymptomatic subjects, but this relationship is inferred *a posteriori*. The unsupervised models are not explicitly designed to perform classification. The specific unsupervised model employed here corresponds to Bayesian factor analysis. This model represents the gene-expression values of each sample in terms of a linear combination of factors. Within the model we impose that each factor is sparse, meaning that only a relatively small fraction of the genes have non-zero expression within the factor loading. This sparseness seeks to map each factor to a biological pathway by identifying genes which are co-expressed, and each pathway is assumed to be represented in terms of a small fraction of the total number of genes. The number of factors appropriate for the data is inferred, using a statistical tool termed the beta process [15]. We have found that, for the virus data considered here, the factor score associated with one of these factors is a good marker as to whether the sample will be symptomatic, but we underscore that this symptomatic/asymptomatic information is not employed in the model.

## Supporting Information

Figure S1 **For the H1N1 Challenge Trial, individual symptom scores of symptomatic infected patients from the time of inoculation (time 0) through the end of the study.**
(PDF)

Figure S2 **Variation over time of the expression of the top 30 individual genes which make up the Influenza factor.**
(PDF)

Figure S3 **Cross-validation of H1N1 (Top) and H3N2 (Bottom) derived factors.**
(PDF)

Figure S4 **Genes comprising the discriminative.** Factor for Influenza infection are involved in canonical antiviral pathways, such as the STAT-1 dependent portions of Interferon-response and dsRNA-induced innate signaling depicted here (top), and the IRF-7 and RIG-I, MDA-5 dependent portions of Interferon-response and ssRNA-induced innate signaling (bottom, www.genego.com). Pathways impacted by genes from the discriminative Factors are marked with a red target symbol.
(PDF)

Figure S5 **Temporal development of the combined Influenza Factor applied to H1N1 (pp top) and H3N2 (bottom) cohorts.**
(PDF)

Figure S6 **Influenza Factor score compared with clinical symptom score over time for all individuals in the study.**
(PDF)

Figure S7 **Performance of the Influenza Factor.** The Influenza Factor develops accurate discriminative utility early in the course of influenza infection, as illustrated by ROC curves for the Factor at each successive timepoint. Depicted are: H1N1-derived Factor applied to H1N1 subjects (A), H3N2 Factor applied to H1N1 subjects (B), H1N1 Factor applied to H3N2 subjects (C), and the H3N2 Factor applied to H3N2 subjects (D).
(PDF)

Table S1 **Patient demographics and pre-challenge serology for HAI titers to challenge viruse (H1N1).** Unique ID’s in Blue indicate ‘symptomatic infected’ individuals.
(PDF)

Table S2 **Patient demographics and pre-challenge serology for HAI titers to challenge viruse (H3N2). Unique ID’s in Blue indicate ‘symptomatic infected’ individuals.**
(PDF)

Table S3 **Complete subject list for both H1N1 and H3N2 viral challenge trials, with total symptom scores and clinical/virologic classifications.**
(PDF)

Table S4 **Comparison of the top 50 genes from the discriminative factors derived from H1N1 and H3N2 challenge trials, ranked by order of individual contribution to the strength of the Factor (highest contributors at the top).**
(PDF)

Methods S1 **Additional material defining the statistical models used are presented.**
(PDF)
