# Supplementary figures and images for: A Host Transcriptional Signature for Presymptomatic Detection of Infection in Humans Exposed to Influenza H1N1 or H3N2
Source: PLoS One. 2013 Jan 9;8(1):e52198. doi: 10.1371/journal.pone.0052198 (PMC3541408; doi:10.1371/journal.pone.0052198)

**Figure s1.** Individual symptom scores of symptomatic-infected patients over time.

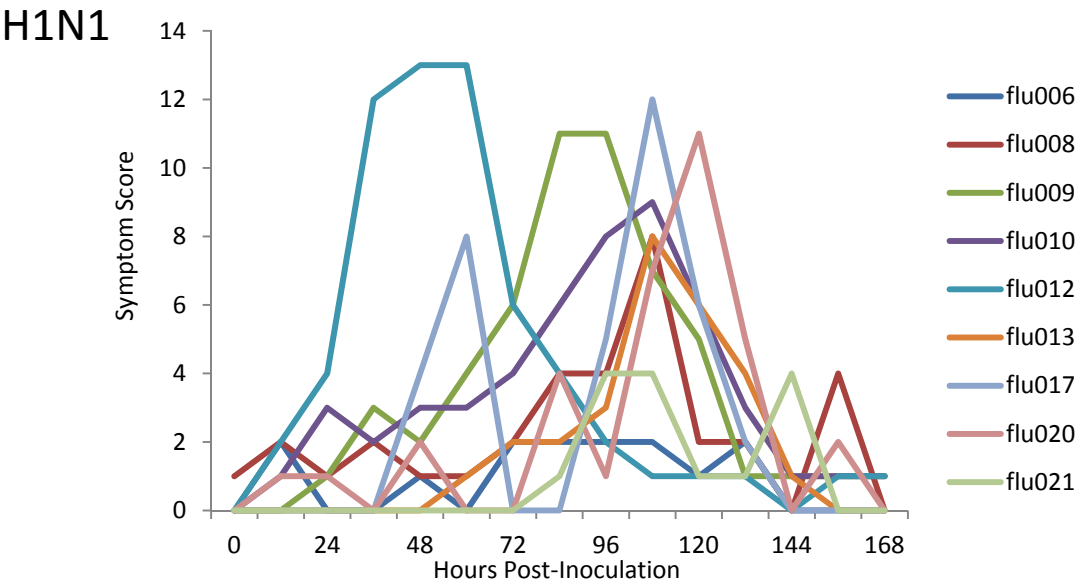

Supplement: Figure S1 — For the H1N1 Challenge Trial, individual symptom scores of symptomatic infected patients from the time of inoculation (time 0) through the end of the study. (PDF) [file pone.0052198.s001.pdf]

**Figure s3.** Cross-validation of H1N1 (Top) and H3N2 (Bottom) derived factors.

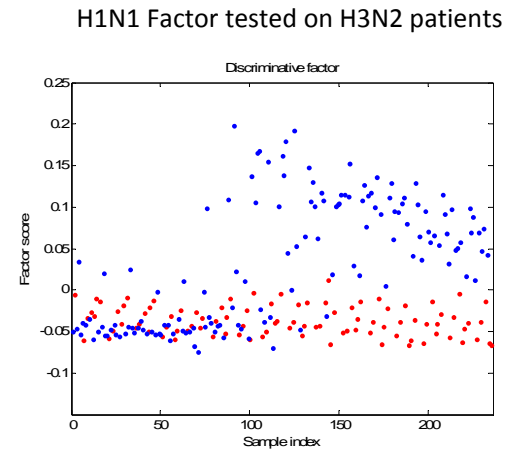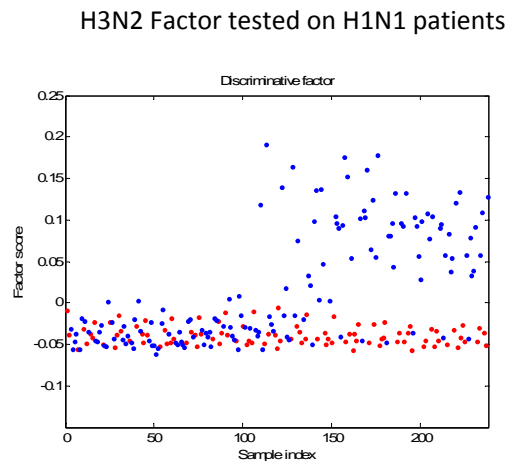

Supplement: Figure S3 — Cross-validation of H1N1 (Top) and H3N2 (Bottom) derived factors. (PDF) [file pone.0052198.s003.pdf]

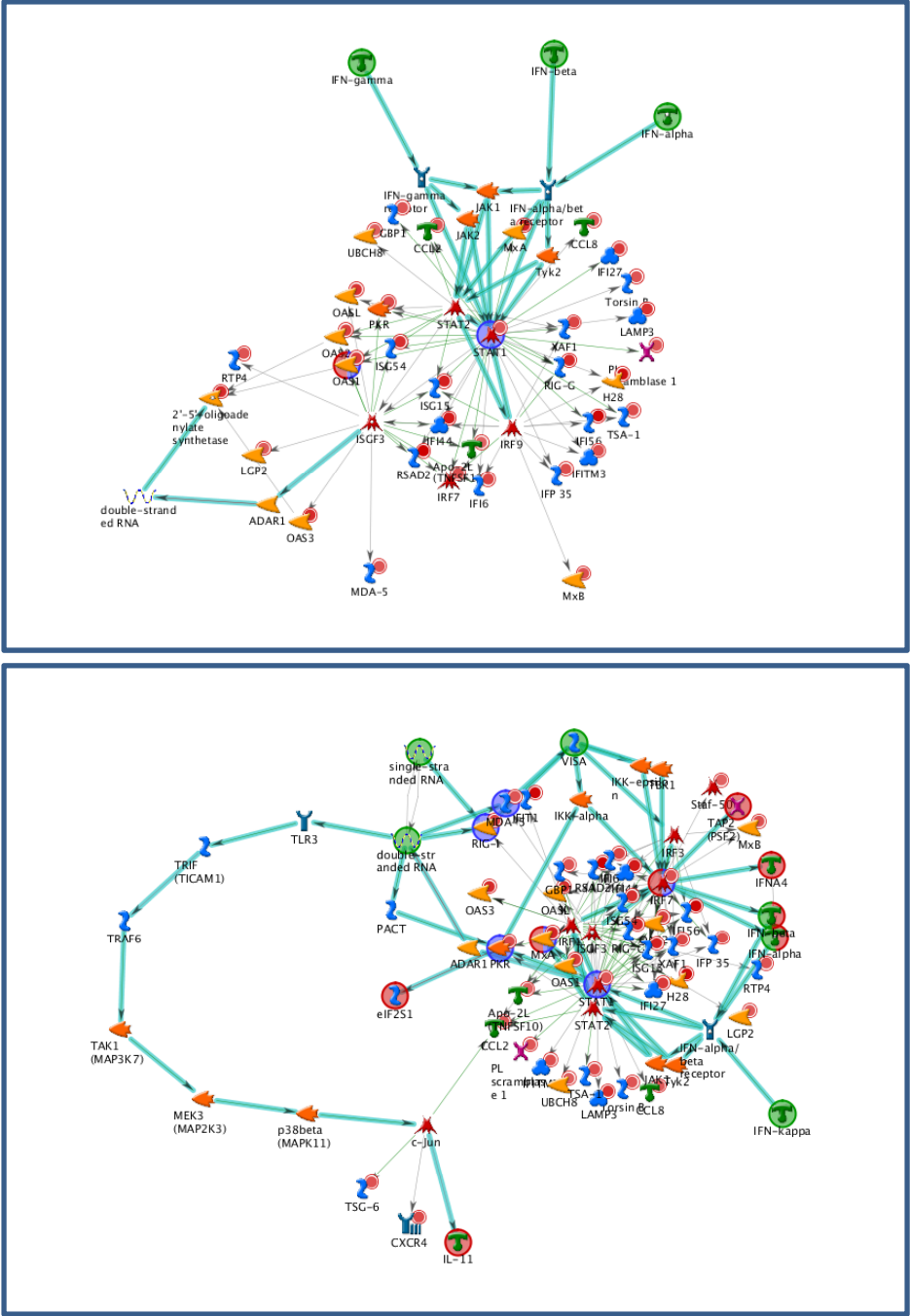

Supplement: Figure S4 — Genes comprising the discriminative. Factor for Influenza infection are involved in canonical antiviral pathways, such as the STAT-1 dependent portions of Interferon-response and dsRNA-induced innate signaling depicted here (top), and the IRF-7 and RIG-I, MDA-5 dependent portions of Interferon-response and ssRNA-induced innate signaling (bottom, www.genego.com). Pathways impacted by genes from the discriminative Factors are marked with a red target symbol. (PDF) [file pone.0052198.s004.pdf]
