# Supplementary material for: A Host Transcriptional Signature for Presymptomatic Detection of Infection in Humans Exposed to Influenza H1N1 or H3N2
Source: PLoS One. 2013 Jan 9;8(1):e52198. doi: 10.1371/journal.pone.0052198 (PMC3541408; doi:10.1371/journal.pone.0052198)

**Figure s2.** Variation over time of the expression of the top 30 individual genes which make up the Influenza factor.

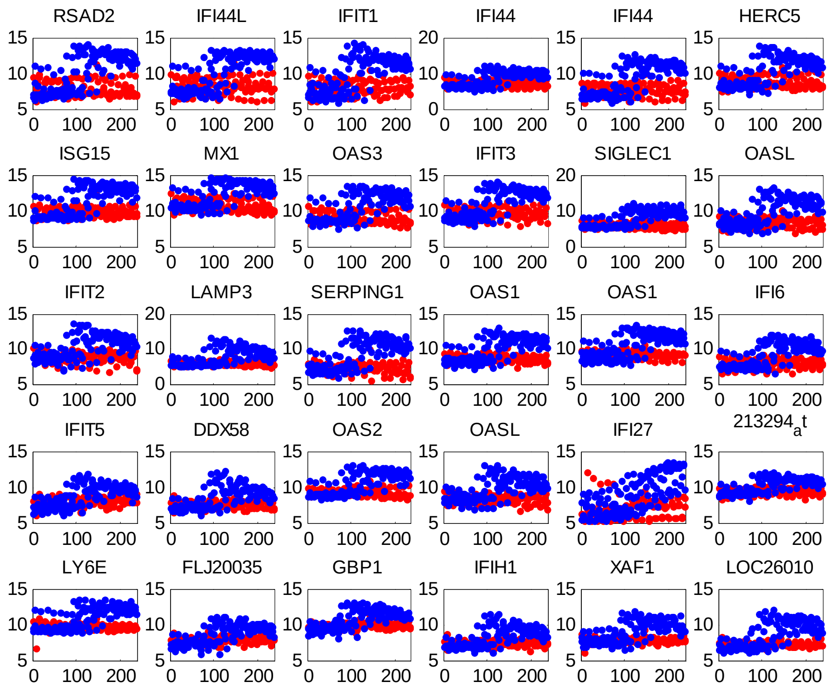

Supplement: Figure S2 — Variation over time of the expression of the top 30 individual genes which make up the Influenza factor. (PDF) [file pone.0052198.s002.pdf]
