# Supplementary material for: A Host Transcriptional Signature for Presymptomatic Detection of Infection in Humans Exposed to Influenza H1N1 or H3N2
Source: PLoS One. 2013 Jan 9;8(1):e52198. doi: 10.1371/journal.pone.0052198 (PMC3541408; doi:10.1371/journal.pone.0052198)

Figure s5. Temporal development of the combined Influenza Factor applied to H1N1 (top) and H3N2 (bottom) cohorts.

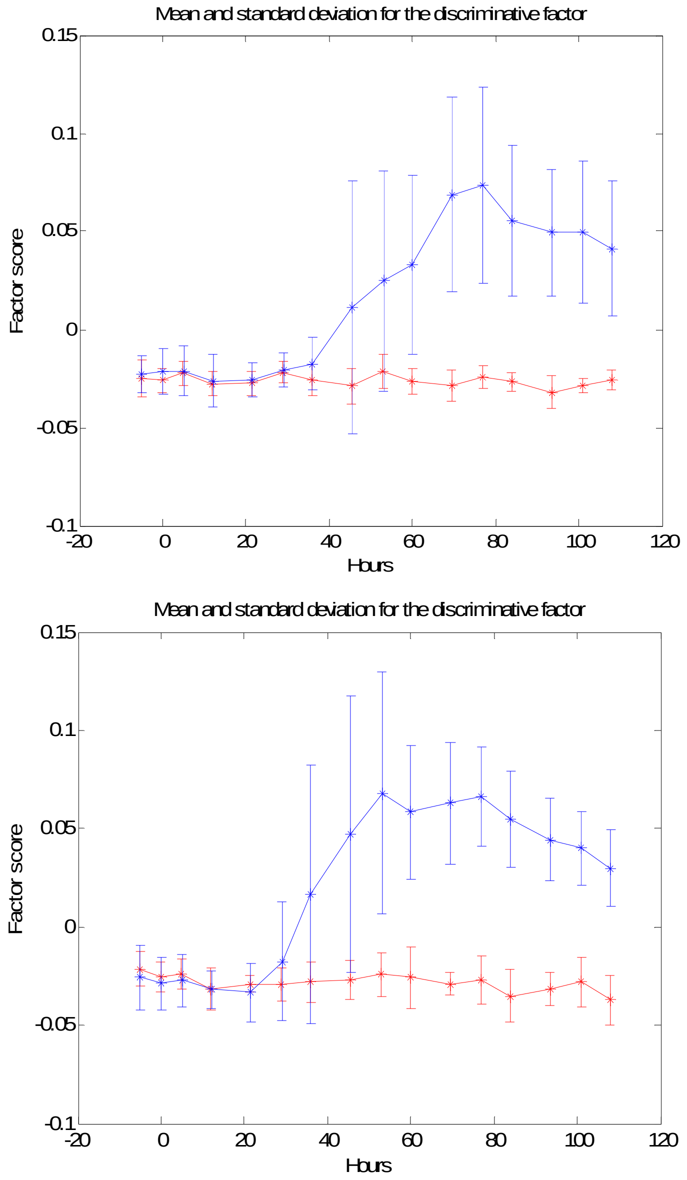

Supplement: Figure S5 — Temporal development of the combined Influenza Factor applied to H1N1 (pp top) and H3N2 (bottom) cohorts. (PDF) [file pone.0052198.s005.pdf]
