# Supplementary material for: A Host Transcriptional Signature for Presymptomatic Detection of Infection in Humans Exposed to Influenza H1N1 or H3N2
Source: PLoS One. 2013 Jan 9;8(1):e52198. doi: 10.1371/journal.pone.0052198 (PMC3541408; doi:10.1371/journal.pone.0052198)

Figure s6. Influenza Factor score compared with clinical symptom score over time for all individuals in the study.

H1N1

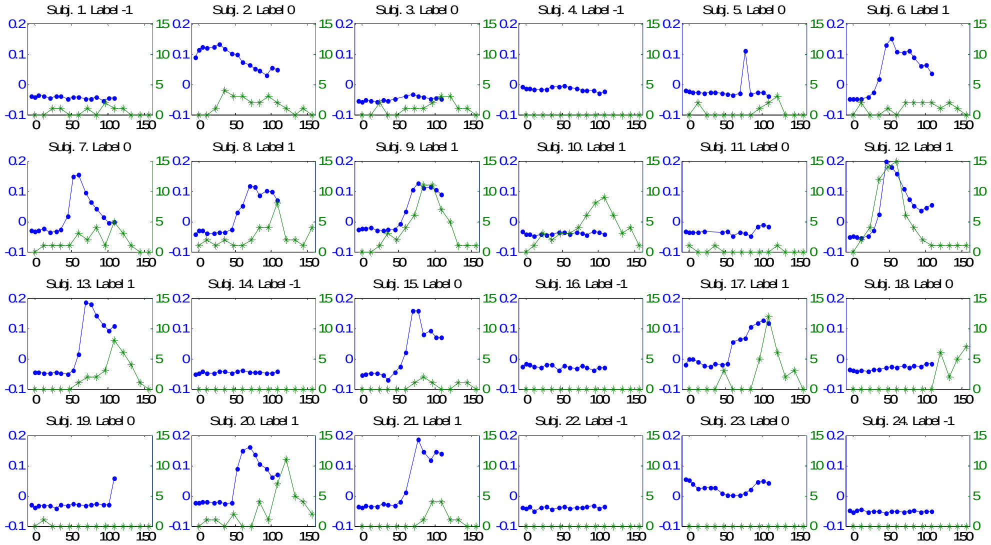

H3N2

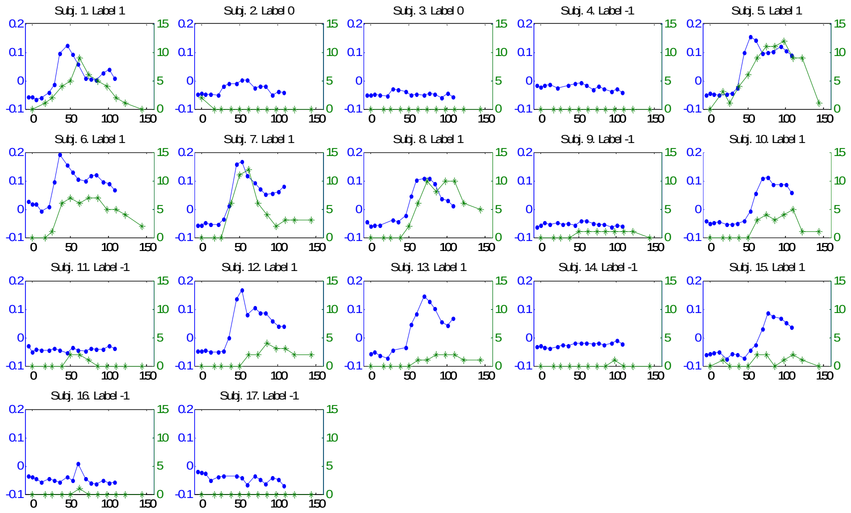

Supplement: Figure S6 — Influenza Factor score compared with clinical symptom score over time for all individuals in the study. (PDF) [file pone.0052198.s006.pdf]
