# Supplementary material for: A Host Transcriptional Signature for Presymptomatic Detection of Infection in Humans Exposed to Influenza H1N1 or H3N2
Source: PLoS One. 2013 Jan 9;8(1):e52198. doi: 10.1371/journal.pone.0052198 (PMC3541408; doi:10.1371/journal.pone.0052198)

Figure s7. Performance of the Influenza Factor. The Influenza Factor develops accurate discriminative utility early in the course of influenza infection, as illustrated by ROC curves for the Factor at each successive timepoint. Depicted are: H1N1-derived Factor applied to H1N1 subjects (A), H3N2 Factor applied to H1N1 subjects (B), H1N1 Factor applied to H3N2 subjects (C), and the H3N2 Factor applied to H3N2 subjects (D).

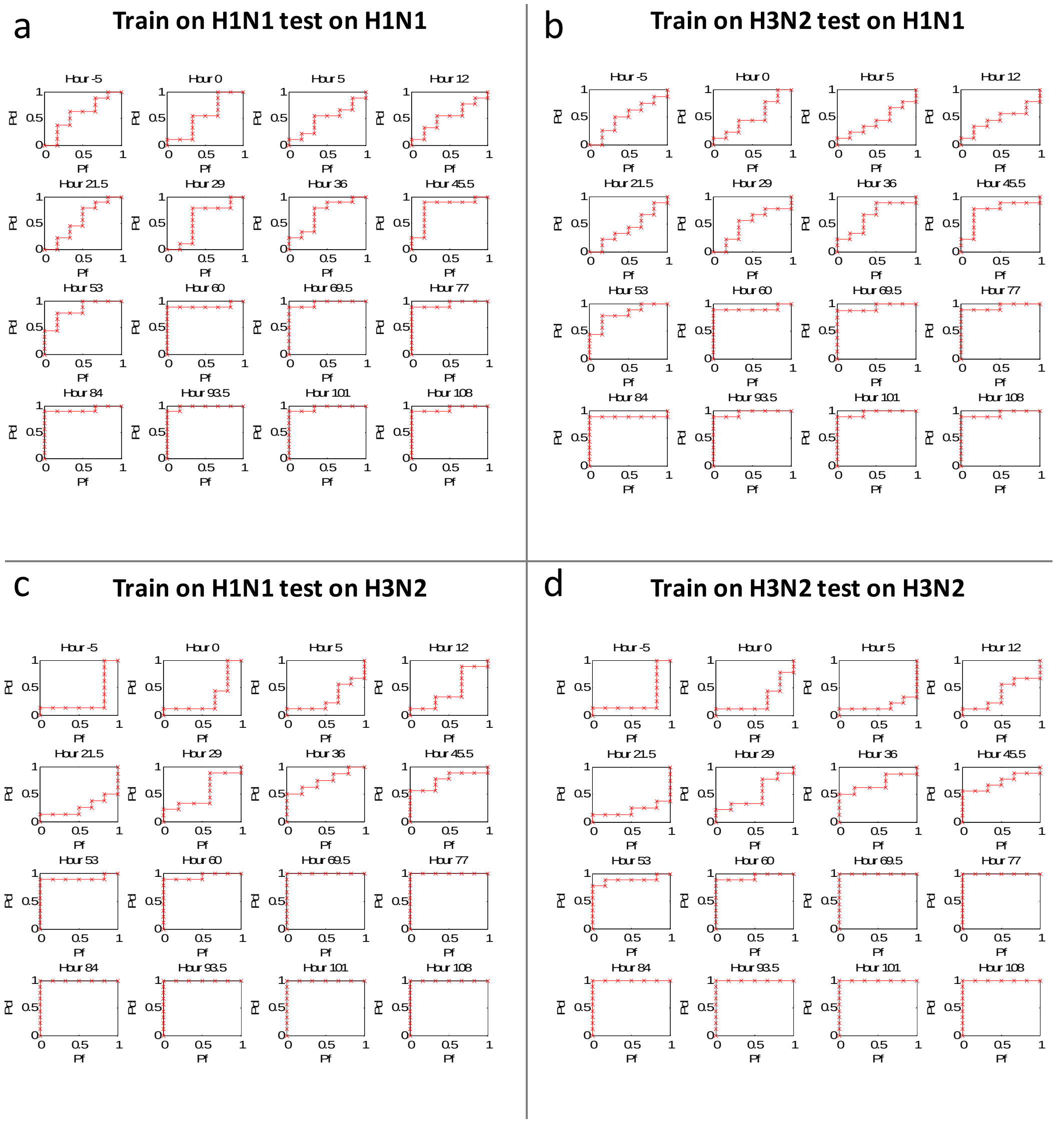

Supplement: Figure S7 — Performance of the Influenza Factor. The Influenza Factor develops accurate discriminative utility early in the course of influenza infection, as illustrated by ROC curves for the Factor at each successive timepoint. Depicted are: H1N1-derived Factor applied to H1N1 subjects (A), H3N2 Factor applied to H1N1 subjects (B), H1N1 Factor applied to H3N2 subjects (C), and the H3N2 Factor applied to H3N2 subjects (D). (PDF) [file pone.0052198.s007.pdf]
