# Supplementary material for: A Host Transcriptional Signature for Presymptomatic Detection of Infection in Humans Exposed to Influenza H1N1 or H3N2
Source: PLoS One. 2013 Jan 9;8(1):e52198. doi: 10.1371/journal.pone.0052198 (PMC3541408; doi:10.1371/journal.pone.0052198)
Supplement: Table S1 — Patient demographics and pre-challenge serology for HAI titers to challenge viruse (H1N1). Unique ID’s in Blue indicate ‘symptomatic infected’ individuals. (PDF) [file pone.0052198.s008.pdf]

*ND – None Detected

**Table s1.** Patient demographics and pre-challenge serology for HAI titers to challenge viruse (H1N1). Unique ID's in Blue indicate 'symptomatic infected' individuals.

| H1N1 | | | | Pre-Screening visit | | Day -1 | |
|---|---|---|---|---|---|---|---|
| Unique ID | Gender | Age (years) | Race/Ethnicity | Date | HAI Titer* | Date | HAI Titer* |
| **flu001** | male | 23 | Caucasian | 6/3/2009 | ND | 8/19/2009 | ND |
| **flu002** | male | 22 | Caucasian | 7/2/2009 | ND | 8/19/2009 | ND |
| **flu003** | male | 20 | Caucasian | 6/23/2009 | ND | 8/19/2009 | ND |
| **flu004** | male | 31 | Caucasian | 6/12/2009 | ND | 8/19/2009 | ND |
| **flu005** | female | 32 | Caucasian | 6/23/2009 | ND | 8/19/2009 | ND |
| **flu006** | female | 24 | Caucasian | 7/2/2009 | ND | 8/19/2009 | ND |
| **flu007** | male | 25 | Caucasian | 6/8/2009 | ND | 8/19/2009 | ND |
| **flu008** | male | 22 | Caucasian | 6/12/2009 | ND | 8/19/2009 | ND |
| **flu009** | male | 35 | Caucasian | 6/19/2009 | ND | 8/19/2009 | ND |
| **flu010** | male | 24 | Caucasian | 6/24/2009 | ND | 8/19/2009 | ND |
| **flu011** | female | 20 | Caucasian | 6/22/2009 | ND | 8/19/2009 | ND |
| **flu012** | female | 24 | Caucasian | 5/29/2009 | ND | 8/19/2009 | ND |
| **flu013** | female | 35 | Caucasian | 6/9/2009 | ND | 8/19/2009 | ND |
| **flu014** | male | 26 | Caucasian | 6/12/2009 | ND | 8/19/2009 | ND |
| **flu015** | male | 24 | Caucasian | 10/2/2009 | ND | 8/19/2009 | ND |
| **flu016** | male | 28 | Hispanic | 6/1/2009 | ND | 8/19/2009 | ND |
| **flu017** | male | 25 | Caucasian/Other | 3/3/2009 | ND | 8/19/2009 | ND |
| **flu018** | male | 23 | Caucasian/Other | 6/10/2009 | ND | 8/19/2009 | ND |
| **flu019** | female | 21 | Caucasian | 6/8/2009 | ND | 8/19/2009 | ND |
| **flu020** | female | 23 | Caucasian | 6/3/2009 | ND | 8/19/2009 | ND |
| **flu021** | male | 20 | Caucasian | 6/29/2009 | ND | 8/19/2009 | ND |
| **flu022** | male | 19 | Caucasian | 6/22/2009 | ND | 8/19/2009 | ND |
| **flu023** | male | 26 | Caucasian | 5/26/2009 | ND | 8/19/2009 | ND |
| **flu024** | male | 27 | Caucasian | 6/26/2009 | ND | 8/19/2009 | ND |
