# Supplementary material for: A Host Transcriptional Signature for Presymptomatic Detection of Infection in Humans Exposed to Influenza H1N1 or H3N2
Source: PLoS One. 2013 Jan 9;8(1):e52198. doi: 10.1371/journal.pone.0052198 (PMC3541408; doi:10.1371/journal.pone.0052198)
Supplement: Table S2 — Patient demographics and pre-challenge serology for HAI titers to challenge viruse (H3N2). Unique ID’s in Blue indicate ‘symptomatic infected’ individuals. (PDF) [file pone.0052198.s009.pdf]

Table s2. Patient demographics and pre-challenge serology for HAI titers to challenge viruse (H3N2). Unique ID's in Blue indicate 'symptomatic infected' individuals.

H3N2				Pre-Screening visit		Day -1	
**Unique ID**	**Gender**	**Age (years)**	**Race/Ethnicity**	**Date**	**HAI Titer***	**Date**	**HAI Titer***
**flu001**	Female	29	Black/Africa Origin	7/23/2008	ND	10/25/2008	ND
**flu002**	Male	28	Caucasian/White	10/9/2008	ND	10/25/2008	ND
**flu003**	Male	24	Caucasian/White	9/22/2008	ND	10/25/2008	ND
**flu004**	Female	23	Caucasian/White	7/16/2008	ND	10/25/2008	ND
**flu005**	Female	25	Caucasian/White	7/16/2008	ND	10/25/2008	ND
**flu006**	Female	28	Mixed Ethnicity	7/3/2008	ND	10/25/2008	ND
**flu007**	Male	35	Caucasian/White	9/19/2008	ND	10/25/2008	ND
**flu008**	Male	25	Caucasian/White	9/22/2008	ND	10/25/2008	ND
**flu009**	Male	24	Caucasian/White	9/15/2008	ND	10/25/2008	ND
**flu010**	Female	22	Caucasian/White	7/3/2008	ND	10/25/2008	ND
**flu 011**	Female	25	Caucasian/White	7/17/2008	ND	10/25/2008	ND
**flu012**	Male	27	Caucasian/White	7/15/2008	ND	10/25/2008	ND
**flu013**	Female	29	Caucasian/White	9/15/2008	ND	10/25/2008	ND
**flu014**	Female	22	Caucasian/White	7/22/2008	ND	10/25/2008	ND
**flu015**	Male	26	Caucasian/White	9/5/2008	ND	10/25/2008	ND
**flu016**	Male	41	Caucasian/White	9/2/2008	ND	10/25/2008	ND
**flu017**	Male	33	Indian	9/15/2008	ND	10/25/2008	ND

*ND – None Detected
