# Supplementary material for: A Host Transcriptional Signature for Presymptomatic Detection of Infection in Humans Exposed to Influenza H1N1 or H3N2
Source: PLoS One. 2013 Jan 9;8(1):e52198. doi: 10.1371/journal.pone.0052198 (PMC3541408; doi:10.1371/journal.pone.0052198)
Supplement: Table S3 — Complete subject list for both H1N1 and H3N2 viral challenge trials, with total symptom scores and clinical/virologic classifications. (PDF) [file pone.0052198.s010.pdf]

Table s3. Complete subject list for both H1N1 and H3N2 viral challenge trials, with total symptom scores and clinical/virologic classifications.

Study	Subject #	Total Sx Score (5 days)	Clinically Symptomatic	Clinically Symptomatic and Microbiologically Infected
H3N2	001	28	Y	Y
	002	0	N	N*
	003	0	N	N*
	004	0	N	N
	005	45	Y	Y
	006	30	Y	Y
	007	31	Y	Y
	008	34	Y	Y
	009	5	N	N
	010	14	Y	Y
	011	4	N	N
	012	12	Y	Y
	013	7	Y	Y
	014	1	N	N
	015	7	Y	Y
	016	1	N	N
	017	1	N	N
H1N1	001	5	N	N
	002	13	Y	N
	003	10	Y	N
	004	0	N	N
	005	5	N	N
	006	7	Y	Y
	007	16	Y	N
	008	12	Y	Y
	009	33	Y	Y
	010	26	Y	Y
	011	2	N	N
	012	34	Y	Y
	013	17	Y	Y
	014	0	N	N
	015	4	N	N
	016	0	N	N
	017	19	Y	Y
	018**	6	N	N
	019	0	N	N
	020	23	Y	Y
	021	6	Y	Y
	022	0	N	N
	023	0	N	N
	024	0	N	N

* EXCLUDED IN SECONDARY ANALYSIS AS SUBJECTS HAD EVIDENCE OF ANTIBODY SEROCONVERSION AT 28 DAYS FOLLOWING STUDY WITH >4 FOLD INCREASE IN INFLUENZA ANTIBODY TITER AS COMPARED TO BASELINE.

** EXCLUDED IN ALL ANALYSES AS SYMPTOMS BEGAN LATE AND WERE FELT TO BE RELATED TO INFECTION ACQUIRED IN THE FACILITY, NOT PRIMARY INFECTION RELATED TO INOCULATION.
