# Supplementary material for: A Host Transcriptional Signature for Presymptomatic Detection of Infection in Humans Exposed to Influenza H1N1 or H3N2
Source: PLoS One. 2013 Jan 9;8(1):e52198. doi: 10.1371/journal.pone.0052198 (PMC3541408; doi:10.1371/journal.pone.0052198)
Supplement: Table S4 — Comparison of the top 50 genes from the discriminative factors derived from H1N1 and H3N2 challenge trials, ranked by order of individual contribution to the strength of the Factor (highest contributors at the top). (PDF) [file pone.0052198.s011.pdf]

Table s4. Comparison of the top 50 genes from the discriminative factors derived from H1N1 and H3N2 challenge trials, ranked by order of individual contribution to the strength of the Factor (highest contributors at the top).

H1N1	H3N2
RSAD2	RSAD2
IFIT1	IFIT1
IFI44L	IFI44L
HERC5	HERC5
IFI44	IFI44
ISG15	OAS3
MX1	MX1
OAS3	IFIT3
IFIT3	ISG15
IFIT2	SERPING1
SIGLEC1	IFIT2
LAMP3	OASL
OASL	LAMP3
SERPING1	IFI6
IFI27	OAS1
IFIT5	OAS2
OAS2	IFI27
DDX58	XAF1
OAS1	SIGLEC1
IFI6	DDX58
XAF1	IFIT5
IFIH1	DDX60
LAP3	IFITM3
LOC26010	LY6E
DDX60	GBP1
HERC6	IFIH1
EIF2AK2	EIF2AK2
LY6E	LAP3
IFITM3	ZCCHC2
ZCCHC2	LOC26010
IFI35	IFI35
ZBP1	IRF7
TNFAIP6	PLSCR1
IRF7	TNFAIP6
PARP12	PARP12
GBP1	RTP4
MX2	HERC6
TOR1B	STAT1
PLSCR1	SCO2
RTP4	ZBP1
SCO2	UBE2L6
CCL8	TNFSF10
STAT1	TRIM22
CCL2	MX2
SAMD9	TOR1B
DHX58	APOL6
UBE2L6	ATF3
TDRD7	CXCL10
TRIM22	CCL2
TREX1	XIST
