# Supplementary material for: A Host Transcriptional Signature for Presymptomatic Detection of Infection in Humans Exposed to Influenza H1N1 or H3N2
Source: PLoS One. 2013 Jan 9;8(1):e52198. doi: 10.1371/journal.pone.0052198 (PMC3541408; doi:10.1371/journal.pone.0052198)
Supplement: Methods S1 — Additional material defining the statistical models used are presented. (PDF) [file pone.0052198.s012.pdf]

SUPPLEMENTAL MATERIAL ON METHODS

The observed data are represented as $\mathbf{X} \in \mathbb{R}^{p \times n}$, where each column corresponds to one of n samples, quantifying the associated gene-expression values for all p genes under investigation. A factor model [1]–[4] is employed. Specifically, the data are assumed to satisfy

$$\mathbf{X} = \mathbf{A}\mathbf{S} + \mathbf{E} \tag{1}$$

where $\mathbf{A} \in \mathbb{R}^{p \times r}$, $\mathbf{S} \in \mathbb{R}^{r \times n}$ and $\mathbf{E} \in \mathbb{R}^{p \times n}$. We assume that the gene-expression data are centered in advance of the analysis; otherwise, there should be an intercept added to the model. Considering the jth sample, $\boldsymbol{x}_j$, corresponding to the jth column of $\mathbf{X}$, the model states that $\boldsymbol{x}_j = \mathbf{A}\boldsymbol{s}_j + \boldsymbol{e}_j$, where $\boldsymbol{s}_j$ and $\boldsymbol{e}_j$ are the jth columns of $\mathbf{S}$ and $\mathbf{E}$, respectively.

The columns of $\mathbf{A}$ represent the factor "loadings", and rows of $\mathbf{S}$ are often called factors. To address the fact that $n \ll p$, we impose a sparseness constraint on the columns of $\mathbf{A}$ [4], with the idea that each column of $\mathbf{A}$ should ideally correspond to a biological "pathway", which should be defined by a relatively small number of correlated genes. Within Bayesian formalisms, the sparse columns of $\mathbf{A}$ are imposed via spike-slab-like priors [4], [5], as discussed further below.

For the factor model in (1), r defines the number of factors responsible for the data $\mathbf{X}$, and it is not known in general, and must be inferred. Within the analysis we consider K *potential* factors (K columns of $\mathbf{A}$), with K set to a value anticipated to be large relative to r. We then infer the number of columns of $\mathbf{A}$ needed to represent the observed data $\mathbf{X}$, with this number used as an estimate of r. Since we will be performing a Bayesian analysis, we will infer a posterior density function on r. Henceforth we assume $\mathbf{A}$ has K columns, with the understanding that we wish to infer the $r < K$ columns that are actually needed to represent the data.

Let $\boldsymbol{a}_k$ represent the kth column of $\mathbf{A}$, for $k = 1, \ldots, K$, and $\boldsymbol{e}_j$ and $\boldsymbol{s}_j$ represent respectively the jth columns of $\mathbf{E}$ and $\mathbf{S}$ (with $j = 1, \ldots, n$). Within the imposed prior, vectors $\boldsymbol{e}_j$ and $\boldsymbol{s}_j$ are generated as $\boldsymbol{s}_j \sim \mathcal{N}(0, \mathbf{I}_K)$, and $\boldsymbol{e}_j \sim \mathcal{N}(0, \text{diag}(\psi_1^{-1}, \ldots, \psi_p^{-1}))$; $\mathbf{I}_K$ is the $K \times K$ identity matrix and the precisions $(\psi_1, \ldots, \psi_p)$ are all drawn i.i.d. from a gamma prior. To define sparseness on the $\boldsymbol{a}_k$ [4] we employ a spike-slab prior:

$$A_{lk} \sim w_{lk}\delta_0 + (1 - w_{lk})\mathcal{N}(0, \alpha_k^{-1}) \quad , \quad w_{lk} \sim \text{Beta}(a, b) \quad , \quad \alpha_k \sim \text{Gamma}(c, d) \tag{2}$$

where (a, b) are selected as to strongly favor $w_{lk} \to 1$, δ_0 is a distribution concentrated at zero, and $l = 1, \ldots, p$.

The Beta Process (BP) is used to infer the number of needed factors r [6]–[9]. Specifically, for each of the K potential factors, there is an associated probability π_k, for $k = 1, \ldots, K$, and a particular data sample utilizes the kth factor with probability π_k, and doesn't use it with probability $1 - \pi_k$ (*i.e.,* Bernoulli). Within the model each of the π_k are drawn

$$\pi_k \sim \text{Beta}(\alpha/K, \beta(K-1)/K) \tag{3}$$

Note that for finite settings of the parameters α and β, this is a degenerate beta distribution [6], which strongly favors $\pi_k \to 0$. Therefore, what this model is imposing is that as the number of potential factors K becomes large, only a small subset of the $\{\pi_k\}_{k=1,K}$ are likely to be large, and therefore only a small subset of factors are utilized to represent the data. The model therefore encourages a parsimonious use of factors for representation of the data; more details may be found in [6]–[9].

The computations are performed using Gibbs sampling, for which all needed conditional density functions are analytic. The results presented here correspond to using 5000 collection samples, after a burn-in of 2000 iterations. However, with 2000 burn-in iterations and 500 collection samples, the average results of the factor scores and factor loadings are almost identical to those found with 5000.

REFERENCES

[1] P. Rai and H. Daum'e III, "The infinite hierarchical factor regression model," in *Proc. Conf. Neural Information Proc. Systems (NIPS)*, Vancouver, Canada, 2008.

[2] D. Knowles and Z. Ghahramani, "Infinite sparse factor analysis and infinite independent components analysis," in *7th International Conference on Independent Component Analysis and Signal Separation*, 2007.

[3] E. Meeds, Z. Ghahramani, R. Neal, and S. Roweis, "Modeling dyadic data with binary latent factors," in *Advances in Neural Information Processing Systems*, 2007, pp. 977–984.

[4] C. Carvalho, J. Chang, J. Lucas, J. R. Nevins, Q. Wang, and M. West, "High-dimensional sparse factor modelling: Applications in gene expression genomics," *Journal of the American Statistical Association*, vol. 103, pp. 1438–1456, 2008.

[5] M. West, "Bayesian factor regression models in the "large p, small n" paradigm," in *Bayesian Statistics 7*, J. M. Bernardo, M. Bayarri, J. Berger, A. Dawid, D. Heckerman, A. Smith, and M. West, Eds. Oxford University Press, 2003, pp. 723–732.

[6] R. Thibaux and M. Jordan, "Hierarchical beta processes and the Indian buffet process," in *International Conference on Artificial Intelligence and Statistics*, 2007.

[7] T. Griffiths and Z. Ghahramani, "Infinite latent feature models and the indian buffet process," in *Advances in Neural Information Processing Systems*, 2005, pp. 475–482.

[8] F. Doshi-Velez, K. Miller, J. V. Gael, and Y. Teh, "Variational inference for the indian buffet process," in *AISTATS*, 2009.

[9] J. Paisley and L. Carin, "Nonparametric factor analysis with beta process priors," in *Int. Conf. Machine Learning*, 2009.
